# Medical Student Leadership Development through a Business School Partnership Model: A Case Study and Implementation Strategy

**DOI:** 10.1177/23821205211010479

**Published:** 2021-04-27

**Authors:** Timothy P Daaleman, Mindy Storrie, Gary Beck Dallaghan, Sarah Smithson, Kurt O Gilliland, Julie S Byerley

**Affiliations:** 1 Department of Family Medicine, University of North Carolina School of Medicine, Chapel Hill, NC, USA; 2 University of North Carolina Kenan-Flagler Business School, Chapel Hill, NC, USA; 3 Office of Medical Education, University of North Carolina School of Medicine, Chapel Hill, NC, USA; 4 Division of General Internal Medicine, University of North Carolina School of Medicine, Chapel Hill, NC, USA

**Keywords:** Leadership development, medical education, curriculum, business school

## Abstract

**Background:**

There is an ongoing call for leadership development in academic health care and medical students desire more training in this area. Although many schools offer combined MD/MBA programs or leadership training in targeted areas, these programs do not often align with medical school leadership competencies and are limited in reaching a large number of students.

**Methods:**

The Leadership Initiative (LI) was a program created by a partnership between a School of Medicine (SOM) and Business School with a learning model that emphasized the progression from principles to practice, and the competencies of self-awareness, communication, and collaboration/teamwork. Through offerings across a medical school curriculum, the LI introduced leadership principles and provided an opportunity to apply them in an interactive activity or simulation. We utilized the existing SOM evaluation platform to collect data on program outcomes that included satisfaction, fidelity to the learning model, and impact.

**Results:**

From 2017 to 2020, over 70% of first-year medical students participated in LI course offerings while a smaller percentage of fourth-year students engaged in the curriculum. Most students had no prior awareness of LI course material and were equivocal about their ability to apply lessons learned to their medical school experience. Students reported that the LI offerings provided opportunities to practice the skills and competencies of self-awareness, communication, and collaboration/teamwork.

**Discussion:**

Adding new activities to an already crowded medical curriculum was the greatest logistical challenge. The LI was successful in introducing leadership principles but faced obstacles in having participants apply and practice these principles. Most students reported that the LI offerings were aligned with the foundational competencies.

## Introduction

Over a decade ago, the Institute of Medicine advocated for academic medical centers to develop leaders at every level who have the capacity to manage the organizational and system changes required to improve health.^1^ The call for leadership development continues today with a heightened emphasis on the integration of medical education and health care service delivery.^2^ In undergraduate medical education, the Association of American Medical Colleges delineates the expectation that graduating medical students will have the ability to “provide leadership skills that enhance team functioning, the learning environment, and/or the health care delivery” as a core competency prior to entering residency training.^3,4^ Medical students, in turn, are consistent in their attitudes that leadership skills are positive contributors to their future roles as physicians,^5^ and most desire more training in this area.^6^

A 2017 survey of the state of leadership development in US medical schools reported that many institutions lack formal curricula and that only one-third of schools require students to participate in a leadership curriculum.^3^ Medical schools that provide elective leadership training programs report low student participation rates, which may reflect lack of student interest, lack of incentives to pursue leadership training, or competing curricular demands.^3^ Several schools offer specialized MD/MBA combined programs^7,8^ or leadership training with special focus in areas such as primary care, population health, or advocacy;^9,10^ however, it is unclear how widely these programs reach across student populations.

A 2014 systematic review of leadership training in undergraduate medical education found that existing curricula included a range of content, modes of delivery, and competencies that were not purposefully aligned with established leadership competency frameworks.^8^ In addition, most evaluations of these training initiatives did not demonstrate meaningful changes in student outcomes.^8^ The review highlighted the importance of aligning proposed leadership curricula with competency models, the need to standardize student outcome evaluations, and the importance of longitudinal integration of curricula across undergraduate medical education.^8^

There is a continued emphasis on leadership development in academic health care systems;^2^ however gaps remain in developing competency-based curricula and in identifying and implementing best practices.^3^ Although other organizations have models and strategies for leadership development that have traditionally looked to business and management education,^11^ business schools have not previously collaborated with medical schools—beyond MD/MBA programs—to develop curriculum that meaningfully engages all medical students.^3,7,8^ In response, we describe the development and initial implementation of a competency-based medical school leadership program, called the Leadership Initiative (LI), that was guided by a business school partnership.

## Leadership Initiative Program

### School of medicine curriculum

The SOM admits 190 students per year and the 4-year curriculum is divided into 3 phases; an 18-month Foundation Phase, 12-month Application Phase, and 14-month Individualization Phase. The Foundation Phase integrates basic sciences and organ systems, clinical skills education, and professional development through 3 courses; Medical Science, Patient Centered Care, and Social and Health Systems. The Application Phase provides integrated clerkship experiences that promote clinical skill building. The longer Individualization Phase provides electives and research opportunities which allow exploration of career options before students apply to residency training programs. In 2015, the SOM was selected to join the second cohort of the AMA Accelerating Change in Medical Education Initiative with a project that focused on developing a leadership curriculum.

### LI program administration and guiding principles

The Leadership Initiative (LI) established an administrative infrastructure and guiding principles first. The education deans from the SOM and BS nominated Leadership Initiative directors from each school who exhibited a collaborative mindset along with content expertise and experience in leadership and professional development. The directors crafted shared goals for the program, which contributed to the following vision statement: to enrich and broaden SOM students’ capacity for ongoing growth in leadership development through an integrated approach that focuses on performance and character, and is grounded in leading-edge academic research and the successful practice of medicine.

The directors adopted the BS learning model that emphasizes the progression from principles to practice, to feedback, and then to reflection. Conceptually, the curricular offerings in the learning model introduce leadership principles, and then provide opportunities to practice leadership in real-world situations and simulations. Learners subsequently receive feedback on their performance, reflect, and then learn from that feedback. Ideally, the learning model strives to have students complete the cycle through curricular and co-curricular activities as many times as possible.

Once the goals, vision statement, and learning model were established, an advisory committee composed of senior education leaders in the SOM, medical students, and administrative support staff was formed. The LI directors scheduled planning sessions for curriculum development, and the output from this work was reviewed by, and input received from, the advisory committee. In addition, the ongoing work of the LI was communicated through existing SOM governance structures, such as the SOM Education Committee.

### Identifying leadership competencies

**Figure 1.**
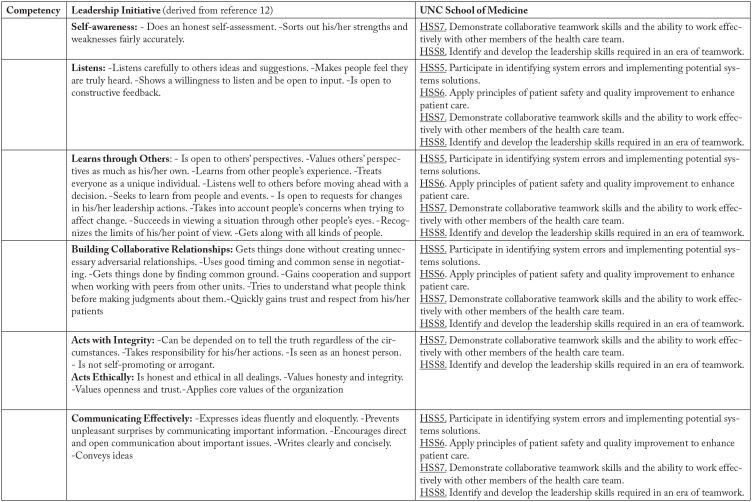
UNC School of Medicine competencies and leadership initiative competencies.

The second phase of LI curriculum development focused on identifying leadership competencies. The LI directors used Values Explorer™, which has been developed by the Center for Creative Leadership (Greensboro, NC) to explore and understand values at the individual and organizational level^12^ as a source tool in developing leadership competencies. The directors independently reviewed the 60-card deck, which designates human values and defining characteristics, and selected cards that were aligned with the SOM competencies and were concordant with the LI vision statement and learning model. The directors then met to review and discuss their card selections until consensus was reached. The consensus selections were member-checked by the advisory committee and are presented in Figure 1.

### Curriculum development and implementation strategy

The identified competencies were used to guide development of LI course offerings in an intentional fashion across the SOM curriculum. Promoting self-awareness and the ability to communicate effectively were foundational competencies that were placed early in the curriculum. The capacity to build collaborative relationships, teamwork skills, and learning through others were also deemed foundational competencies and located throughout subsequent phases of the SOM curriculum. Finally, the competencies of leading ethically and with integrity were targeted to the Individualization Phase in Year 4.

The next period of development and implementation focused on identifying opportunities in the SOM curriculum for delivering content and providing educational activities that were aligned with the competencies. The medical school LI director met individually with SOM course directors to provide an overview of the LI, and to explore areas where the LI could complement and add value to existing courses. The strategy, in a packed curriculum, focused on identifying ways to add LI content and activities to existing material in the curriculum. Course directors in the health system science course (SHS), as well as the required residency development experience (Transition to Residency), identified curricular needs and invited the LI team to develop offerings for their respective courses.

Once these opportunities were identified, the LI directors worked with BS and SOM faculty to develop instructional material and approaches for delivering content. The output from this developmental work was shared iteratively with SHS course directors for further refinements. Table 1 presents the LI curriculum offerings with brief descriptions of the learning activity, the source content, and linkages to the enabling competencies. The StrengthsFinder, SBIA (Situation, Behavior, Impact, Action), and TeamSTEPPS activities were embedded in the required SHS 1 and 2 curricula, and Peak Performance was placed in SHS 5. StrengthsFinder and Peak Performance were piloted in the first year of the LI rollout. In the second year, SBIA, TeamSTEPPS, and the Challenge Course, a team-building activity piloted during the Transition to Residency experience in the fourth year to students matching in neuroscience and family medicine residency programs, were added. The SBIA activity was cancelled in 2018 due to a hurricane.

**Table 1.** Leadership initiative curriculum offerings.

Enabling competency	Content source	Location in curriculum	Learning activity
Increase self-awareness	Strengths Finder (SF)	Foundation PhaseSHS 1	-Strengths Finder book introduced and distributed during orientation.-Students asked to complete online self-assessment and bring report to small group session.-Interactive activity on recognizing strengths in self and others in small group.
Improve communication skills; giving and receiving effective feedback	Situation/behavior/impact/action (SBIA) feedback model	Foundation PhaseSHS 1	-Pre-class work of reviewing SBIA model and identifying a feedback situation to practice.-Small group activity of role play using identified pre-class situations.-Rotating roles as provider and receiver of feedback, and observer who shares insights.
Refine communication skills; enhance giving and receiving effective feedback skills; develop teamwork skills	Team STEPPSChallenge course	Foundation PhaseSHS 3Individualization PhaseTransition to residency	-Large group theory burst introducing Team STEPPS concepts.-Small group interactive activities including paper airplane factory and helium stick.-Large group debrief.-A facilitated series of interactive and team-based activities, in which participants learn about themselves, each other, and the group as a unit.
Increase self-awareness; enhance personal performance; refine communication skills; manage conflict	Peak performance simulation	Individualization PhaseSHS 5	-Computer-based simulation of upper level resident on hospital service who must address series of team, interpersonal, and patient communications in real time.-Small group debrief with clinician and executive coach.-Optional completion of reflection workbook and personal development plan.

SOM faculty who facilitated the LI offerings underwent a training session before each of the respective activities. The activities also leveraged both content expertise and resources from the BS and SOM, such as faculty from the Institute for Healthcare Quality Improvement and the Outdoor Education Center. The Peak Performance simulation, which has been described in more detail separately,^13^ utilized executive coaches from BS as well as clinical SOM faculty who underwent training prior to the activity.

### LI program evaluations

The program evaluation and associated data collection and reporting protocols were reviewed by the institutional Office of Human Research Ethics, which determined that it did not require IRB approval. We utilized the existing SOM evaluation platform to collect data on LI program outcomes from 2017 to early 2020 for StrengthsFinder and SBIA. For TeamSTEPPS, the Challenge Course, and Peak Performance we designed an electronic evaluation that was independent of the SOM platform. These activities are offered and evaluated in the spring semester and we did not have data for 2020 offerings due to COVID-19. Evaluations included an open text field that allowed students to provide comments.

## Results

First year students participating in the course included 178 (94%) in 2017 to 2018, 140 (74%) in 2018 to 2019, and 187 (98%) in 2019 to 2020. For fourth year students in the Peak Performance offering, 18 (9%) participated in the 2017 to 2018 pilot program, which was gradually expanded to 110 (58%) in 2018 to 2019.

We used the Net Promoter Score (NPS) to gauge the level of overall satisfaction with designated LI offerings (Table 2). The NPS is a marketing industry index that assesses how likely customers are to recommend a brand or product to others (scale 1-10).^14^ The NPS (average 6.1) indicated that students were not particularly satisfied with the learning experience in these activities.

**Table 2.** Leadership initiative program evaluation.

Course offering	N	Item: Were you aware of content before this activity? Yes (%)	Item: I will be able to immediately apply this learning to my medical school experience. Mean Score*	Item: The learning environment reflected respect for students’ opinions, questions, and comments. Mean Score*	Net promoter score. Item: How likely is it that you would recommend this experience to your classmates? Mean Score^!^
Strengths^#^ Finder	327	94 (29)	3.25	4.45	6.0
SBIA^@^	171	31 (18)	3.6	4.5	6.6
Team STEPPS^&^	138	16 (12)	3.4	4.2	5.8

* Scale: 1 = strongly disagree to 5 = strongly disagree.

# 2018-2019, 2019-2020; ^@^2019-2020; ^&^2018-2019.

! Scale: 1 = not at all likely to 10 = extremely likely.

Although no formal qualitative methods were used, the LI directors reviewed student comments and reached consensus regarding illustrative comments. Participants suggested 3 potential reasons for the limited satisfaction. The first may be considered as structural elements (eg, source material, course logistics) of the activity and were represented by, “It was interesting to see my own strengths, how those strengths compared to others, and the commonalities seen between everyone in the class. However, I also feel as if this activity was kind of wedged in between other classes.” Process elements of the activity, such as how course content was delivered, were another component and typified by, “I did not enjoy the role playing. I thought it was not very helpful in terms of future situations where feedback is essential.” The final element was varying levels of participant resistance to the activity and were characterized by, “the exercises did not respect the time of the students” and “we know who we are and the skills we have.”

To determine fidelity of the LI learning model (ie, introduce principles and provide opportunity to practice) in this initial phase of program development, we used evaluation items from the StrengthsFinder, SBIA, and TeamSTEPPS activities. We asked participants if they were aware of the source material before the activity to gauge if we were presenting new principles and content. A minority of students (20%) reported prior knowledge of course material. Participants were also asked about their level of agreement using a scale of 1 to 5 (1 = strongly disagree to 5 = strongly agree) for the following statements: “I will be able to immediately apply this learning to my medical school experience,” and; “the learning environment reflected respect for students’ opinions, questions, and comments.” Students reported that they were equivocal about their ability to immediately apply the leadership material (3.2, SD = 0.2), however agreed that the learning environment was respectful (4.4, SD = 0.15) (Table 2).

We assessed the impact of the LI on promoting specified leadership competencies by asking participants if the activity offered the opportunity to practice the skills and competencies of self-awareness, communication, and collaboration/teamwork (Table 3). A majority of students reported that the StrengthsFinder, SBIA, TeamSTEPPS, and Challenge Course allowed them to practice these foundational competencies. Most students reported that Peak Performance promoted communication competencies, which reflects the offering’s specific design in developing communication skills. Across all activities, participants reported the capacity to practice additional skills and competencies, such as managing conflict, managing others, and openness.

**Table 3.** Leadership initiative competency skill practice.

Course offering	Self-awareness* N (%)	Communication* N (%)	Collaboration* N (%)
Strengths Finder	327 (100)	229 (70)	174 (53)
SBIA	109 (64)	139 (81)	125 (73)
Team STEPPS	44 (32)	61 (35)	61 (35)
Challenge course	22 (50)	27 (61)	29 (66)

* Yes response to “activity offered the opportunity to practice this skill and competency.”

## Discussion

To our knowledge, this is the first report that describes the development and initial implementation of a medical school leadership program that utilized a competency-based model and was guided by a business school partnership. There were several lessons learned which can be categorized as logistical challenges, the learning model, and LI competency alignment.

### Logistical challenges

The greatest logistical challenge we faced was adding a series of new activities to an already crowded medical curriculum. The longitudinal integration of leadership training across a medical school curriculum has been promoted as a solution to the problem of competing demands.^8^ We took a longitudinal design into account when planning the LI by looking to assimilate offerings in the existing social and health system science course (SHS), which threads through the SOM curriculum. The first-year offerings were required courses in the SHS1 and 2 curricula which would account for the high rate of participation. The lower participation rate in the fourth-year reflects pilot work of these courses with a lesser number of students.

From the inception of the LI and throughout its developmental phase we had the support of SOM leadership and subsequently received buy-in from SHS course directors. This support was critical and led to LI offerings being embedded in existing SHS course scheduling, and designation as a required activity, which are reflected in high participation rates. However this requirement may have also contributed to the restricted level of satisfaction reported with the activities. Students may have perceived the LI activities as supplementary and “one-off” to the SHS course material.

Although we had the support of SHS course directors, the LI was dependent upon a wider group of SOM faculty. We provided training sessions for these faculty using existing SHS faculty meetings, and training sessions that were scheduled immediately before the activity. In these meetings and sessions, SOM faculty expressed uneven levels of support for the LI offerings and varying degrees of engagement with the preparation work. Our mitigation strategy focused on framing LI offerings as complementary to the small group building that was already ongoing in the SHS, and as a value-added faculty development activity.

### LI competency alignment

Most students reported that the offerings were aligned with the LI foundational competencies. There is minimal literature describing competency-based leadership frameworks and models.^8^ The Medical Leadership Competency Framework (MLCF) has been developed by the National Health Service to promote leadership development for medical students and practicing physicians in the United Kingdom (UK).^15^ The MLCF competencies, which include demonstrating personal qualities, working with others, setting direction, and managing and improving services,^15^ share characteristics with the Leadership Initiative (LI) competencies of self-awareness, learning through others, communicating effectively, and building collaborative relationships.

Although the MLCF and LI competencies are comparable, the development process for each respective competency set was different. The MLCF process involved a comprehensive literature review of medical leadership, comparative analysis of leadership competency frameworks, and iterative consultations and input from medical practice and education stakeholders in the UK.^15^ In contrast, the development process for the LI competencies utilized resources and collaboration from business and management education, which may provide a more expedited approach that can be individualized to existing educational environments and curricula. Although strategies for implementing the MLCF have not been systematically described and evaluated,^15^ a qualitative study reported that medical students had conflicting views about when to introduce the content but felt that they had to have some clinical experience in order to appreciate the importance of leadership.^16^

Three competency-based leadership curricula have been previously described; none have been based in US medical schools.^8^ An elective leadership rotation in the UK focused on 3 domains in the MLCF framework (ie, setting direction, managing and improving services) utilizing a shadowing experience with a hospital administrator.^17^ The CanMEDs framework, a model that identifies competencies for all areas of medical practice in Canada,^18^ was used to guide the curriculum for an emergency medicine global health elective in Toronto.^19^ Finally, a 1-week leadership training course in Sweden was guided by 2 Accreditation Council for Graduate Medical Education competencies (ie, interpersonal and communication skills, systems-based practice) and used a module design with experienced-based learning and reflection.^20^

### Limitations

Although this initiative was undertaken at a single institution, we have detailed the process by which this leadership initiative was developed, which could be replicated at other medical schools. We did not conduct a process evaluation to identify and monitor barriers and facilitators to the logistical process of implementing the LI curriculum. An additional limitation was that our evaluation data measured outcomes only related to the effectiveness of each respective LI offering. Finally, since this study describes the initial implementation period of individual LI activities, we will need to examine the cumulative impact of the entire LI program across a cohort of medical students.

## Conclusion

The LI is a competency-based medical school leadership program that was guided by a business school and developed in an intentional and progressive fashion. The learning model introduces leadership principles and ideally provides opportunities to practice these principles in activities and simulations. Self-awareness, communicating effectively, and teamwork/collaborative relationships were foundational competencies that were situated early in the curriculum. The LI program and implementation strategy is feasible and may help inform emerging leadership curricula development in undergraduate medical education.

The evaluation of our initial implementation demonstrated mixed results and points to areas of improvement and adaptation for this program and other emerging curricula. Although our curricular content was competency based and well planned, future strategies that promote greater integration with the SOM core curriculum will be necessary to enhance greater sustained student engagement with the material. Students will also need opportunities to practice and reinforce leadership skills, and to receive meaningful feedback on their performance to see the relevance of this content across their medical education. While clear that physicians need effective skills in health systems science, inspiring students to incorporate these skills, in addition to the nationally assessed basic science and clinical knowledge based content, will be challenging going forward.^21^ Medical schools must continue to refine methods of curricular integration geared toward producing physicians optimally prepared to serve in the modern health care environment.
